# The Microstructure and Properties of CoCrFeNi/WC-Nb HEA Composite Coating Prepared by Laser Cladding

**DOI:** 10.3390/ma19132866

**Published:** 2026-07-04

**Authors:** Haihong Fan, Zijian Liu, Haomu Zhu, Liancai Pang, Jiang Huang

**Affiliations:** 1 College of Electronics and Information Engineering, Guangdong Ocean University, Zhanjiang 524088, China; 2 Guangdong Provincial Key Laboratory of Intelligent Equipment for South China Sea Marine Ranching, Guangdong Ocean University, Zhanjiang 524088, China

**Keywords:** laser cladding, HEA, microstructure, in situ reaction, corrosion resistance, wear resistance

## Abstract

CoCrFeNi/WC-Nb high-entropy alloy (HEA) composite coating was prepared on the surface of Q235 steel by LC (laser cladding) technology, and the effects of WC and in situ NbC reinforcement on the coating were studied. The phase composition, phase characteristics, microhardness, and wear resistance of the cladding coatings were characterized by scanning electron microscope (SEM), X-ray diffraction (XRD), friction and wear tester, and X-ray photoelectron spectroscopy (XPS), and the corrosion resistance was tested by a three-electrode electrochemical workstation. The results show that the CoCrFeNi/WC-Nb HEA coating consists of FCC, WC, NbC, and Laves phases, and the reinforcing phase causes grain refinement and lattice distortion. The microhardness reached (418.29 ± 16.72) HV, which was about 2.64-times higher than that of the CoCrFeNi HEA coating. The wear rate decreased to (1.150 ± 0.11) × 10^−4^ mm^3^N^−1^m^−1^, which was about 0.25 times that of the CoCrFeNi HEA coating, and the wear of the coating changed from abrasive wear to adhesive wear. The corrosion current density and corrosion voltage of the CoCrFeNi/WC-Nb HEA coating are (3.3820 ± 0.2103) × 10^−6^ A/cm^2^ and −(0.7650 ± 0.0850) V, respectively.

## 1. Introduction

In 2004, the concept of HEA was put forward for the first time in material science, which broke the original design idea of solute and solvent [1]. HEA is composed of five elements with equal molar ratio [2]. With the deepening and development of HEA research, it can be seen that HEA can also be composed of three or more elements with a near molar ratio. The material structure is stable and tends to form a solid solution, and it has good fatigue resistance, wear resistance, and corrosion resistance, which has aroused great interest and the widespread concern of researchers [3,4,5,6]. With the deepening and development of research, three principal-element HEAs, four principal HEAs, and even multi-principal-element HEAs all have high-entropy effects, thus greatly enriching the design system of HEAs [7,8].

However, HEAs also have many problems, such as the high cost of block manufacturing and difficulty in practical engineering applications. By preparing the HEA coating, researchers can not only obtain coatings with excellent mechanical properties, but also control the engineering cost reasonably, which provides a feasible scheme for the application of HEAs. At present, there are mainly laser metal deposition [9,10], vacuum sintering [11,12], thermal spraying [13], MIG/TIG welding [14], and LC [15,16,17] methods to prepare HEA coatings, among which LC has the advantages of fast processing speed, rapid solidification, firm combination of coating and substrate, and low dilution rate, and is used for the surface modification of various complex structural components. It has been widely used for surface modification and repair [18]. By properly designing HEA components and adding a reinforcing phase, the performance of HEAs can be further optimized and excellent coatings can be prepared. For HEAs, due to their many components and great physical and chemical differences, LC technology can balance the physical and chemical differences of multiple components well to obtain high-quality coatings.

Among many known HEA systems, Cantor-type HEA mainly has a FCC structure (such as CoCrFeNi, CoCrFeMnNi, CoCrFe, etc.) with outstanding ductility, stable mechanical properties at high temperatures, and good corrosion resistance in corrosive environments, and is considered as a HEA with great research significance [19]. Z. Y. Wang et al. [20] prepared a CoCrFeNi HEA coating with LC technology on the surface of TA15 steel, and studied the influence of laser process parameters on the microstructure and wear properties of the coating. The results show that when the power is 650 W, the coating has only a single FCC phase and good wear resistance. When the power is increased to 800 W, brittle TiO_2_ is formed in the coating, which further reduces the wear resistance of the coating. Q. Zhu et al. [21] prepared a CoCrFeNi HEA coating on AISI1045 steel. By annealing the coating to inhibit phase separation and element segregation, a composite coating with uniform element distribution was obtained, thus improving its corrosion resistance. The existing results show that the hardness and wear resistance of CoCrFeNi HEA coatings are not outstanding.

In recent years, the research on directly or indirectly (mainly in situ synthesis) adding a reinforcing phase to improve the properties of the coating has been widely studied. J. Huang et al. [22] added different proportions of Fe-based amorphous alloy to CoCrFeNi HEA powder to prepare crack-free CoCrFeNi HEA/Fe composite coating. The results show that the phase composition of the coating should not change with the increase in Fe-based amorphous alloy, but the microstructure of the coating gradually evolved from regular cellular crystals to irregular amorphous structures. When x = 8%, the corrosion current density and wear rate of the composite coating are the lowest. S.Q. Cao et al. [23] prepared a CoCrFeNi-TiAl composite coating on the surface of 316 stainless steel. It was found that after adding TiAl, the microstructure of the coating changed from a single FCC long columnar structure to a FCC + BCC biphasic eutectic structure, and the content of the eutectic structure was also closely related to the proportion of added components. Coherent precipitation and the dislocation synergistic effect of the L2_1_ phase lead to the remarkable improvement of the mechanical properties of the composite coating. A large number of research results show that adding ceramic phases, such as WC, TiC, and NbC, is a feasible method to enhance the mechanical properties of coatings [24,25,26,27]. However, the brittleness of ceramic carbide and the mismatch between atomic size and coating powder often lead to cracking of the coating, which limits the practicality of the reinforcing phase.

Therefore, based on Q235 steel, this study prepared Co and CC composite coatings on its surface by LC technology. By adding Nb to this strong carbon-forming element, NbC was formed by an in situ reaction to inhibit brittle cracking caused by the direct addition of WC, and the coating properties were improved. The research results can provide useful guidance for the preparation of high-performance HEA composite coatings by combining direct addition and in situ synthesis.

## 2. Experimental Procedures

### 2.1. Material Preparation

**Figure 1:**
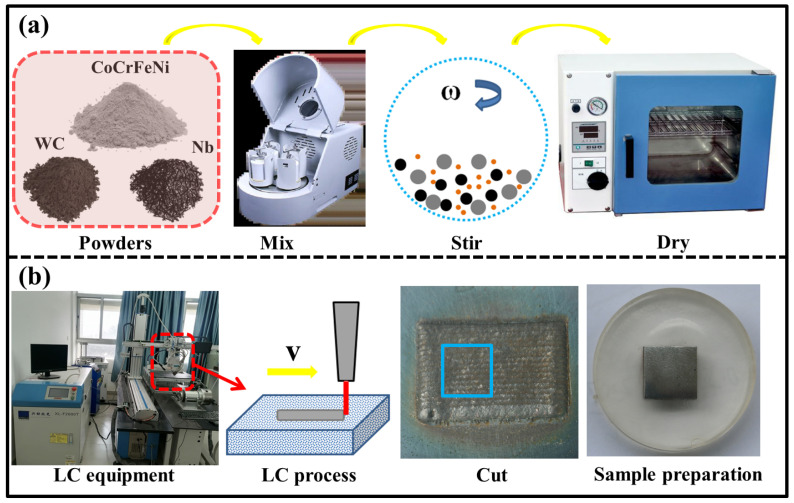
The schematic diagram of powder preparation and sample preparation. (**a**) Powder preparation process, (**b**) Coating preparation process.

In this experiment, Q235 was selected as the substrate, and its size is 100 × 50 × 3 mm (length × width × height). Use sandpaper to polish the surface of the substrate, remove the rust and stains on the surface, wash with anhydrous ethanol, and dry for later use. The powder was poured into a planetary mixer and stirred for 2 h, and then dried in a drying oven at 50 °C for 6 h. The fully stirred mixed powder was made into a 1 ± 0.1 mm thick coating using a standard mold. The average particle sizes of CoCrFeNi HEA, WC, and Nb powders were 80–125 μm, 100–125 μm, and 40–65 μm, respectively. The chemical properties of the Q235 and powders are shown in Table 1. Figure 1a shows the powder preparation process, while Figure 1b shows the LC sample preparation process. The purpose of this work is to study the synergistic enhancement of the direct addition of ceramic particles and in situ synthesis of ceramics relative to CoCrFeNi HEA coatings, so a CoCrFeNi HEA coating (marked as HEA) and a CoCrFeNi HEA/WC-Nb (70% CoCrFeNi, 20% WC and 10% Nb, mass fraction) composite coating (marked as HEA/WC-Nb) were prepared, respectively.

The XL-FW2000 fiber laser processing system (Guangzhou Xingrhenium Laser Equipment Co., Ltd., Guangzhou, China) was used in the experiment. According to the preliminary work [28,29] and a large number of pre-experiments, the parameters used were set as shown in Table 2. During the experiment, the whole laser head and coating processing area were wrapped with an acrylic board and maintained with argon gas throughout. After the coating was prepared, it naturally cooled to room temperature under argon protection. The coating was cut into 10 × 10 mm samples by a wire-cutting machine, and then the structure and mechanical properties were analyzed.

### 2.2. Phase and Morphology Characterization

The phase composition of the sample was obtained using a XRD (XRD, SmartLab 9 KW, Rigaku, Tokyo, Japan) diffractometer at 40 kV and 40 mA; the radiation source was Cu-K_α_. The scanning angle range was 20–90°, and the scanning speed was 2°/min. The microstructure and wear morphology of the cross-section of the samples were analyzed by SEM (SEM, FEI, QUANT 250, Eindhoven, The Netherlands) with an energy dispersion spectroscope (EDS, Noran System 7, Thermo Fisher Scientific, Waltham, MA, USA) to assist in analyzing the distribution characteristics of elements in point scanning or mapping. XPS (XPS, Thermo K-Alpha, New York, NY, USA) was used to test the chemical properties of the surface after the electrochemical experiment.

### 2.3. Microhardness, Wear, and Corrosion Resistance

The microhardness of the cross-section of the sample was completed by a Vickers hardness tester (MHVD-1000AT, Shanghai, China); the load was 200 g and the dwell time was 10 s. From the substrate to the coating, the interval between each test was is 0.1 mm, and three parallel points were measured and averaged. The wear properties of the coatings were tested at room temperature by using a pin-disc friction wear tester (SFT-2M, Lanzhou, China). The indenter was a 3 mm Si_3_N_4_ ceramic ball with a load of 30 N and a rotational radius of 2 mm, the rotational speed was 200 r/min, and the test duration was 30 min. At room temperature, the corrosion resistance of the sample was tested by using a three-electrode electrochemical workstation (CS350M, Wuhan CorrTest Instruments Co., Ltd., Wuhan, China). The electrolyte was a 3.5 wt.% NaCl solution, and the surface area of the coating was 1 cm^2^. Before the test, immerse the sample for 24 h to stabilize the open circuit potential (OCP). The potential range of the potential polarization test was in the range of −1.5–0.5 V, the scanning rate was 1 mV/s, and the electrochemical impedance spectroscopy (EIS) was set at a frequency range of 10^−1^–10^5^ HZ. In order to ensure the reliability of the experimental data, the friction, wear, and electrochemical tests were repeated three times under the same conditions and averaged.

## 3. Results and Discussion

### 3.1. Phase Analysis of the Samples

**Figure 2:**
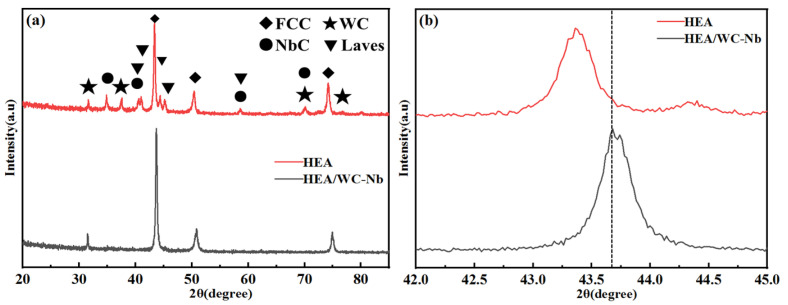
XRD patterns of HEA and HEA/WC-Nb coatings. (**a**) All-region XRD, (**b**) Local XRD.

**Figure 3:**
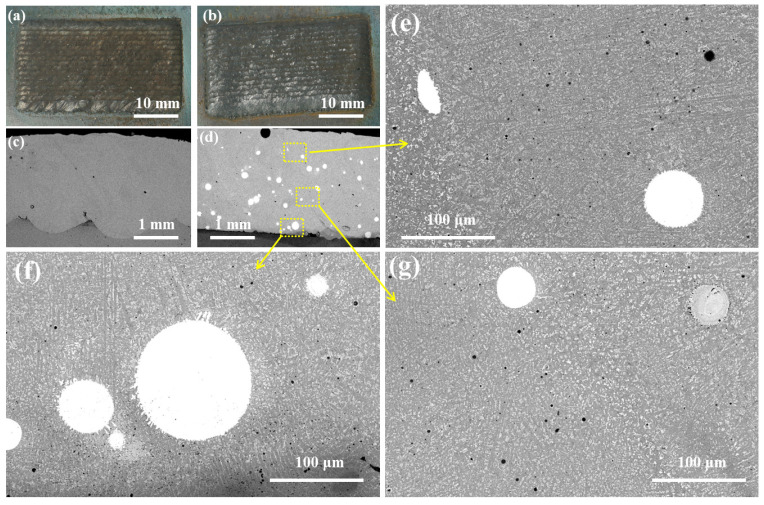
The surface and cross-sectional morphology of the following coatings: (**a**) HEA; (**b**) HEA/WC-Nb. (**c**–**g**) SEM of each designated area.

Figure 2 shows the XRD patterns of HEA and HEA/WC-Nb coatings. As shown in Figure 3a, the HEA coating is a single FCC phase, while the HEA/WC-Nb coating has evolved into a combination of FCC, Laves, WC, and NbC phases. NbC’s PDF card number is PDF#38-1364, which is consistent with the references [30]. The corresponding peak positions are 34.7°, 40.3°, 58.3°, and 69.7°. Among them, the peak positions of 40.3° and 58.3° overlap with Laves, and the peak position of 69.7° overlaps with WC. Under the irradiation of the high-temperature laser, part of the WC in the HEA/WC-Nb coating is decomposed into W and C elements. As Nb is a strong carbide-forming element, Nb and W compete to absorb the C element; thus, in situ NbC is generated [31]. The value of the mixing heat is a measure of the atomic binding capacity. The values of Nb and C are −120 KJ/mol, and the values of W and C are −60 KJ/mol. Obviously, C will take precedence over Nb. It was found that the diffraction peak position of the HEA/WC-Nb coating obviously shifted to the left, and the diffraction peak became wider. According to the Bragg equation, the left shift of the peak position indicates that the lattice spacing increases, and the complex phase combination leads to the increase in the lattice strain, which leads to lattice distortion. The lattice strain is calculated by the Williamson–Hall (W-H) plot method; it can be expressed as [32]:

(1) $$\beta_{T}\cos\theta =\varepsilon (4\sin\theta +\frac{K\lambda }{D})$$

where $\beta_{T}$ is full width at half maximum of the diffraction peak, θ is the Bragg diffraction angle, ε is the lattice strain, K is the shape factor, λ is the wavelength of the X-ray under Cu-K_a_ radiation, and D is the lattice size. Table 3 is obtained by fitting the diffraction peaks of the two samples. According to Equation (1), the lattice strain ε of the HEA and HEA/WC-Nb is calculated to be $1.38\times 10^{-3}$ and $2.49\times 10^{-3}$, respectively. The calculation results show that the in situ NbC, WC, and Laves phases lead to large lattice distortion.

### 3.2. Microstructure Analysis of the Samples

Figure 3 shows the surface and cross-sectional morphology of the HEA and HEA/WC-Nb coatings. Figure 3a,b shows that the coating is well-formed, and there are no cracks and pores. Figure 3c,d is firmly combined, indicating that the experimental parameters are set reasonably. Due to the Marangoni convection effect and the action of gravity, high-density WC (density is about 15.7 g/cm^3^) particles are mainly distributed in the middle and bottom of the coating [33], as shown in Figure 3e,f.

**Figure 4:**
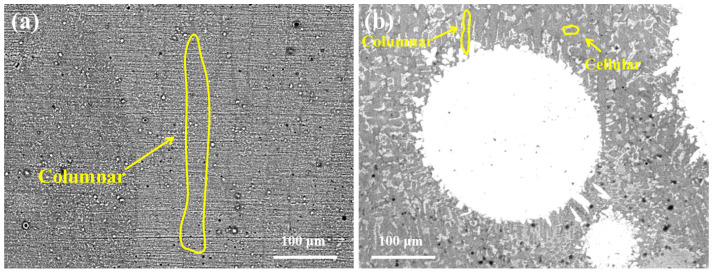
Cross-sectional morphology of the following coatings: (**a**) HEA; (**b**) HEA/WC-Nb.

WC particles have a significant effect on the growth type of coating grains, as shown in Figure 4. When the coating is pure HEA powder, the coating is mainly composed of columnar crystals with a large size. When WC particles are added, the columnar grains of the cladding layer are hindered, and some small-scale columnar and cellular crystals are formed around WC particles, and the columnar grains are almost perpendicular to WC particles. The reason is attributed to the temperature gradient effect [34]. In the micro-molten pool formed by the laser, when the liquid melt solidifies, the heat preferentially flows to the low-temperature, unmelted WC particles, forming micro-directional growth, refining the structure and forming grain refinement and strengthening.

**Figure 5:**
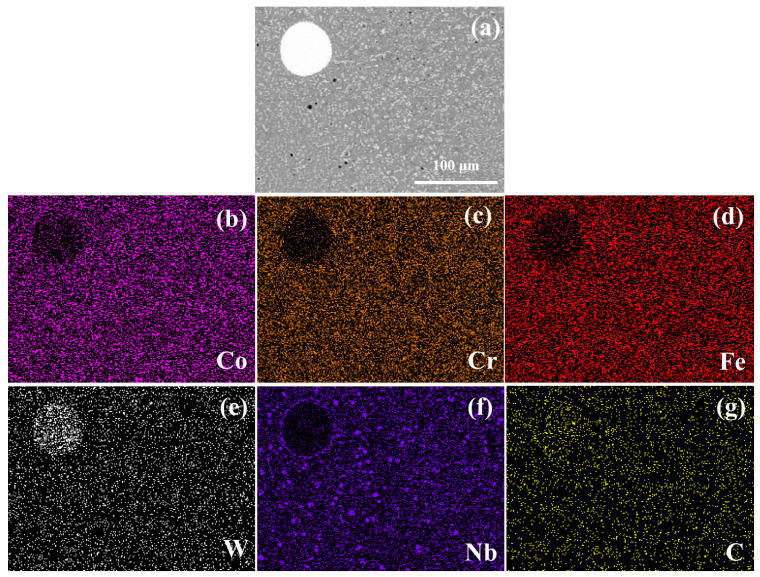
(**a**) SEM and (**b**–**g**) EDS map scanning results of HEA/WC-Nb coatings.

Figure 5 shows the SEM and EDS element distribution of the HEA/WC-Nb coating. Obviously, the white part is WC particles (as shown in Figure 5a,e,g), and the Co, Cr, and Fe elements (as shown in Figure 5b–d) of the HEA are evenly distributed in the area, except the WC particles. Interestingly, the Nb element diffuses outward around the edge of the WC particles, as shown in Figure 5f. This phenomenon corresponds to the dissolution, decomposition, and diffusion of WC particles, and the process of combining Nb with C to create in situ NbC.

### 3.3. Microhardness Analysis of the Samples

**Figure 6:**
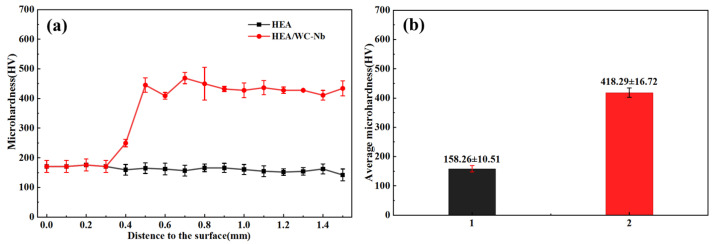
(**a**) Microhardness distribution of HEA and HEA/WC-Nb coatings and (**b**) average microhardness of HEA and HEA/WC-Nb coatings.

Figure 6a,b show the cross-sectional microhardness distribution of samples on the substrate and the average microhardness values of the HEA coating and HEA/WC-Nb coating. The average microhardness of HEA is (158.26 ± 10.51) HV, while that of HEA/WC-Nb is (418.29 ± 16.72) HV, which is about 2.64 times that of HEA. The microhardness test of the coating reveals the close correlation between microhardness difference and structure of the coating. The phase composition and lattice distortion, and lattice size of the coating, affect the microhardness of the coating. Firstly, WC and NbC in the HEA/WC-NbC coating are hard ceramic carbides, which are beneficial to the increase in coating microhardness [35]. However, in this experiment, we did not prepare samples with WC and Nb added independently, so we could not get the specific effects of WC and NbC on the microhardness of the coating. Therefore, the influence of WC and NbC on coating microhardness in this work cannot be fully understood, and further research is needed. Secondly, the lattice size and yield strength of materials are expressed by the Hall–Petch formula as [18,31]:

(2) $$\sigma_{y}=\sigma_{0}+Kd^{-1/2}$$

where $\sigma_{y}$, $\sigma_{0}$ are the yield strength and deformation resistance; K, d are the influence coefficient and the average lattice size. Small-sized in situ NbC particles and Laves phase are formed in the HEA/WC-Nb coating, which leads to the increase in dislocation at the grain boundaries. Under the action of external microstructure and high-temperature force, dislocations hinder the sliding of grain boundaries, and plastic deformation resistance increases, which increases the material’s ability to resist surface morphology and plays a role in grain refinement and strengthening [18]. Thirdly, lattice distortion is positively related to lattice strain. The calculation results in Table 2 show that the lattice strain of the HEA/WC-Nb coating is 1.8 times that of the HEA coating. The strengthening effect of lattice distortion further improves the microhardness of the HEA/WC-Nb coating [36].

### 3.4. Friction and Wear Analysis

**Figure 7:**
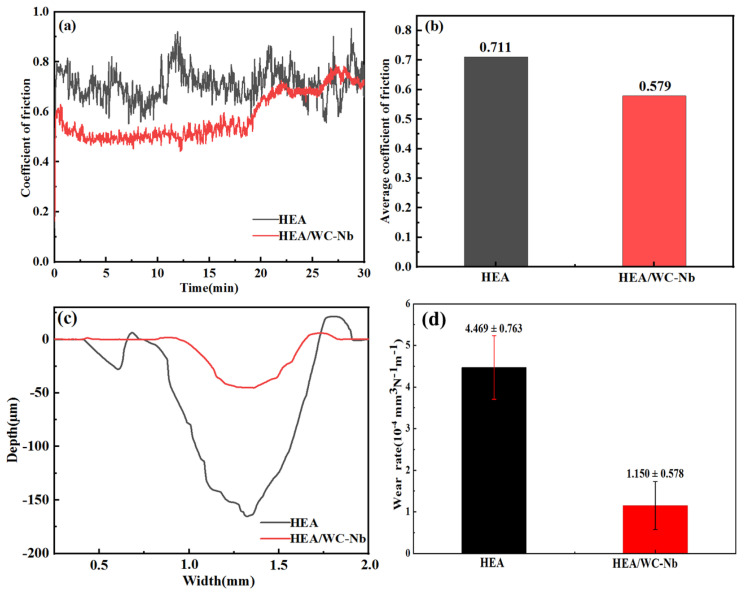
Wear performances of HEA and HEA-WC/Nb coatings. (**a**) Coefficient of friction (COF) curves; (**b**) average COFS; (**c**) cross-sectional profiles; (**d**) wear rates.

Figure 7a shows the COF curves and average COFs of HEA and HEA/WC-Nb coatings sliding against the Si_3_N_4_ ceramic ball. COFs increased sharply at the beginning of the friction experiment, which was caused by the small contact area between the friction ball and the coating and the sinking of the indenter at the initial stage of wear [37]. After the wear time reached 20 min, the COF of the HEA/WC-Nb coating increased, which may be attributed to the oxidation of the sample under friction. Figure 7b shows that the average COF values of HEA and HEA/WC-Nb coatings are 0.711 and 0.579, respectively; the HEA/WC-Nb coating has a lower COF. The wear profiles of two samples were measured by a scanning probe, as shown in Figure 7c. Compared with the HEA/WC-Nb coating, the wear profile of the HEA coating is wider and deeper. The wear rate is calculated by Archard’s equation [38]:

(3) $$L=\frac{V}{N\times d}$$

where V (mm^3^), N (N), and *d* (m) are the wear volume, load, and the total sliding distance. The calculation result is shown in Figure 7d; the wear rates of HEA and HEA/WC-Nb coatings are (4.469 ± 0.763) × 10^−4^ and (1.150 ± 0.578) × 10^−4^, respectively. The wear rate of the HEA/WC-Nb coating is about 0.25 times that of the HEA coating. In conclusion, the HEA/WC-Nb coating has a lower COF, shallower wear profile, and lower wear rate, showing better wear resistance.

**Figure 8:**
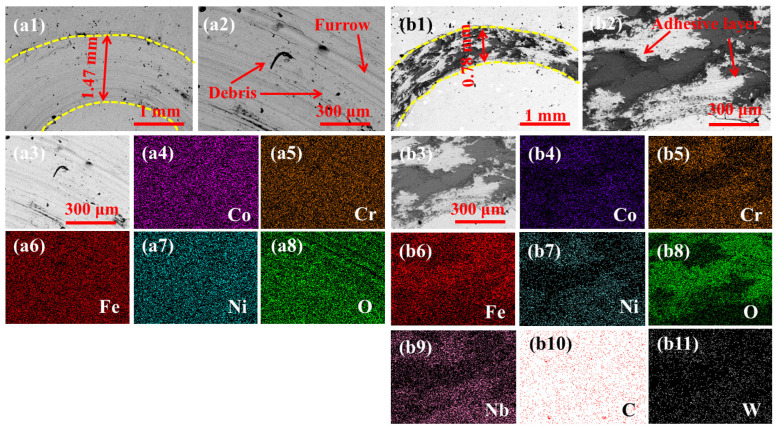
Wear morphology and EDS surface scanning results of the coatings. (**a1**–**a8**) HEA; (**b1**–**b11**) HEA/WC-Nb.

Figure 8(a1–b11) are the wear morphology and EDS surface scanning results of the HEA and HEA/WC-Nb coatings. Figure 8(a1) shows that the wear width of the HEA coating is 1.47 mm, which is about twice that of the HEA/WC-Nb coating (Figure 8(b1)). There are obvious furrows and debris in the wear coating of the HEA (Figure 8(a2)), which shows abrasive wear. However, there are adhesive layers on the HEA WC-Nb coating (Figure 8(b2)), and the wear type is adhesive wear [22]. Figure 8(a3–a8) are the EDS surface scanning images of the wear in HEA micro-areas. The results show that the elements of Co, Cr, Fe, and Ni are evenly distributed without segregation, while the content of O element in the furrow is lower, because oxidized abrasive particles peeled off during the friction process. The surface scanning of the HEA/WC-Nb micro-area (as shown in Figure 8(b3–b11)) showed an obvious segregation phenomenon. It can be seen that the adhesion layer in Figure 8(b3) is seriously oxidized, and the content of Fe is high, while the contents of Nb and W elements in other parts are high, which can effectively inhibit the oxidation of the coating and reduce the generation of brittle oxides, thus improving the wear resistance of the coating.

**Figure 9:**
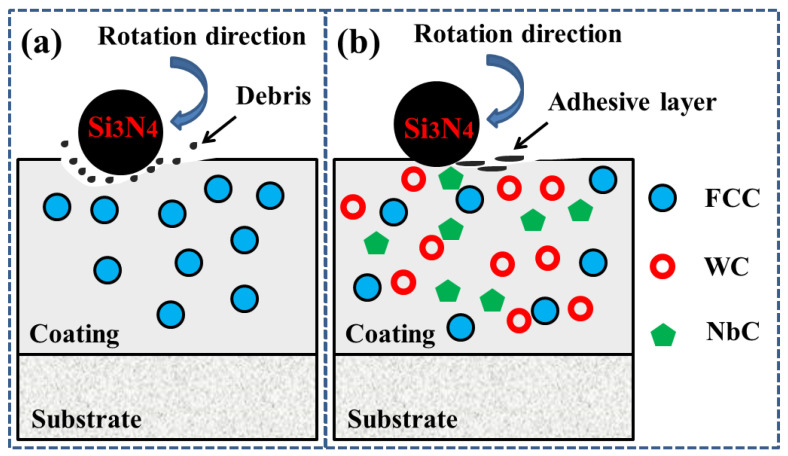
Wear mechanism diagrams of the coatings. (**a**) HEA; (**b**) HEA/WC-Nb.

Figure 9 explains the wear resistance mechanism of the HEA/WC coating by combining the XRD, microstructure, and microhardness results in this study. Firstly, the HEA coating contains only the FCC phase, as shown in Figure 9a, while the HEA/WC-Nb coating contains WC and in situ NbC, both of which are ceramic-reinforced phases with high microhardness. Generally speaking, hardness is directly proportional to wear resistance [39,40]. Therefore, the addition and generation of a reinforcing phase is beneficial to improve the wear resistance of HEA/WC-Nb coatings. Secondly, the HEA/WC-Nb coating has refined grains and larger lattice distortion. Refined grains make the coating denser, while large lattice distortion increases the dislocation energy of atomic sliding, making the coating more stable, thus increasing the wear resistance.

### 3.5. Corrosion Resistance Analysis

**Figure 10:**
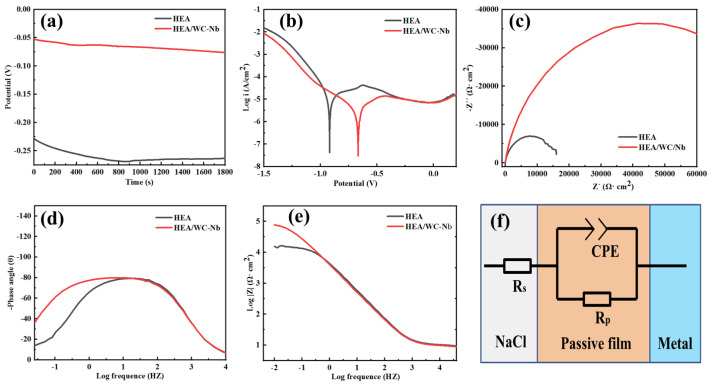
Corrosion electrochemistry of the HEA and HEA/WC-Nb coatings: (**a**) OCP; (**b**) Tafel polarization curves; (**c**) Nyquist impedance spectra; (**d**) Bode plots of phase angle vs. frequency; (**e**) Bode plots of |Z| vs. frequency; (**f**) the equivalent circuit.

Before testing the corrosion resistance, the HEA and HEA/WC-Nb samples were immersed in a 3.5 wt.% NaCl solution for 24 h and then the open circuit voltage (OCP) was tested for 1800 s. The test results are shown in Figure 10a. Both samples obtained stable OCP results. In comparison, the OCP of the HEA/WC-Nb sample is higher, which indicates that its passivation film has stronger protective properties and effectively reduces corrosion sensitivity [41].

In order to evaluate the corrosion resistance of HEA and HEA/WC samples, the Tafel extrapolation method was used to calculate the corrosion potential (E_corr_), corrosion current density (I_corr_), and annual corrosion rate (V_corr_) of the two samples, as shown in Figure 10b. The results are shown in Table 4. E_corr_ is a thermodynamic category, indicating the trend of corrosion. I_corr_ is a kinetic concept, which determines the electrochemical corrosion rate of the coating. V_corr_ is the annual corrosion rate. According to the formula [42,43]:

(4) $$V_{corr}=\frac{Ai_{corr}}{nF}$$

(5) $$i_{corr}=nFv$$

where *A* is the atomic weight, *n* is the valence, *F* is the Faraday constant, and *v* is the electrode surface reaction rate. A higher E_corr_ and lower I_corr_ mean the corrosion resistance of the coating is better. The results in Table 4 show that the corrosion resistance of the HEA/WC-Nb sample is better than that of the HEA sample.

Figure 10c shows the impedance spectra of the coatings. In the impedance spectrum, the capacitance arc is a semi-circular arc, and the radius of the arc reflects the charge transfer resistance on the electrode surface. The larger the arc, the greater the charge transfer resistance. The increase in transfer resistance leads to the decrease in corrosion current, and the sensitivity of the coating to solution corrosion decreases, thus showing higher corrosion resistance [42,44]. Obviously, the HEA/WC-Nb coating has a larger radius and the transfer resistance is much greater than the HEA coating [42]. Figure 10d–e show the Bode spectra of the coatings. Figure 10d is the Bode spectrum of the sample EIS, and the potential range of the test is −0.8–0.2 V. It shows that with the increase in test potential, the phase angle of the Bode spectrum first increases and then decreases, reaching the maximum phase angle in the middle- and low-frequency region, and the maximum phase angle is less than 90 degrees, indicating that the impedance spectrum has ideal capacitive characteristics. Figure 10e shows that the resistance modulus |Z| in the low-frequency component increases. A large phase angle indicates that the passivation film is more stable and dense, and a large resistance modulus means that the penetration of ions on the coating surface is more difficult [43]. In summary, the Nyquist and Bode diagrams are consistent with the test results of the polarization curve. The HEA/WC-Nb coating has better corrosion resistance than that of the HEA coating.

**Figure 11:**
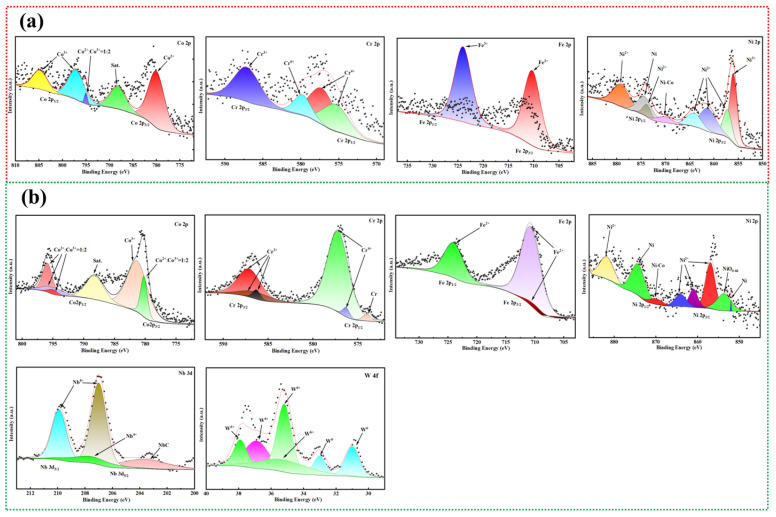
The XPS analysis of the following coatings: (**a**) HEA; (**b**) HEA/WC-Nb.

Considering that ion exchange and passive film formation are inevitable during electrochemical testing, the surface of the sample after electrochemical testing was analyzed by XPS, so as to obtain the mechanism of how W and Nb elements in the HEA/WC-Nb coating adjust the corrosion resistance process. Figure 11a,b show the XPS results of HEA and HEA/WC-Nb coatings, respectively. XPS analysis uses Advantage 9.0 software. The results show that by comparing the elements of Co, Cr, Fe, and Ni in the two coatings, it is found that their peak positions are very close and their contents are relatively consistent. In the HEA/WC-Nb coating, W exists in the form of metals W, W^2+^, and W^6+^, and Nb exists in the form of Nb^2+^ and Nb^4+^. From the above results, a possible explanation for the corrosion resistance mechanism is obtained: Firstly, because the states of Co, Cr, Fe, and Ni in HEA and HEA/WC-Nb coatings are the same, the difference of corrosion resistance between the two coatings is mainly attributed to the compounds formed by W and Nb elements. Secondly, Nb can promote heterogeneous nucleation, refine alloy grains, reduce the number of component segregation and micro-galvanic corrosion, make the distribution of corroded micro-batteries more uniform, and avoid the rapid expansion of local pitting corrosion. Finally, W is often compounded with Nb and other elements, which can improve the overall passivation stability of the alloy and reduce the tendency of pitting corrosion in the 3.5% NaCl solution. The alloy containing W-Nb can show much lower pitting corrosion sensitivity than that added by a single element.

This work shows the synergistic effect of double ceramic carbides as far as possible, but the quantitative analysis of coating properties by NbC and WC is still inconclusive. This problem needs to be further discussed in the follow-up study.

## 4. Conclusions

By using laser cladding technology, 20 wt.% WC and 10 wt.% Nb elements were added to CoCrFeNi HEA and a crack-free HEA composite coating was prepared on the surface of Q235 steel. The main conclusions are summarized as follows:

1. XRD results show that the CoCrFeNi HEA/WC-Nb coating mainly consists of a FCC phase, WC phase, in situ NbC phase, and Laves phase. The addition of phases makes the main peak shift to the left, and grain refinement and lattice distortion increase. The SEM results show that after adding WC and Nb elements, the surrounding of the reinforced phase is mainly composed of small columnar and cell grains.
2. After adding WC and Nb elements, the microhardness of the composite coating reaches 418.29 ± 16.72 HV, which is 2.64-times higher than that of the CoCrFeNi HEA coating.
3. The average wear rate of the CoCrFeNi HEA/WC-Nb coating is (1.150 ± 0.578) × 10^−4^ mm^3^N^−1^m^−1^,which is about 0.25 times of that of the HEA coating. HEA coating mainly shows abrasive wear, while the CoCrFeNi HEA/WC-Nb coating mainly shows adhesive wear. The electrochemical test results show that the corrosion current density of the CoCrFeNi HEA/WC coating is (3.3820 ± 0.0415) × 10^−6^ A/cm^2^ and the corrosion potential is −(0.7650 ± 0.0337) V; thus, the corrosion resistance is effectively improved.

## Figures and Tables

**Table 1:** Chemical composition of the materials used in the experiment.

Material	C	Co	Cr	Fe	Ni	W	Nb	S	Mn	Si	P
Q235B	0.15	-	-	Bal.	-	-	-	0.11	0.25	0.15	0.12
CoCrFeNi	-	26.16	22.98	24.79	26.07	-	-	-	-	-	-
WC	6.12	-	-	-	-	93.88	-	-	-	-	-
Nb	-	-	-	-	-	-	100	-	-	-	-

**Table 2:** Processing parameters of the LC.

Power	Scanning Speed	Spot Radius	Overlapping Ratio	Argon Flow Rate	Defocus
1500 W	500 mm/min	2.5 mm	40%	6.0 L/min	+5 mm

**Table 3:** Corresponding parameters of the Williamson–Hall (W-H) plot method (rad).

Sample	No.	θ	FWHM (β_T_)	β_T_ cos θ	4 sin θ
HEA	1	0.27518	0.00432109	0.00415	1.08687
	2	0.381439	0.00629505	0.00584	1.48903
	3	0.443119	0.0108978	0.00985	1.71502
	4	0.654084	0.00766042	0.00608	2.43372
HEA/WC-Nb	1	0.276248	0.0029524	0.00284	1.09099
	2	0.30436	0.0058671	0.00559	1.19873
	3	0.327255	0.00513493	0.00486	1.28576
	4	0.353722	0.00692006	0.00649	1.38555
	5	0.3580	0.00627202	0.00587	1.40158
	6	0.379382	0.00785276	0.00729	1.48139
	7	0.380262	0.00556079	0.00516	1.48465
	8	0.394315	0.00706876	0.00653	1.5367
	9	0.647584	0.00931604	0.00743	2.41304

**Table 4:** Corrosion parameters and equivalent circuit fitting parameters of HEA and HEA/WC-Nb coatings in the 3.5 wt.% NaCl solution.

Sample	E_corr_ (V)	I_corr_ (A/cm^2^)	V_corr_ (mm/a)
HEA	−(0.9202 ± 0.0520)	(2.9893 ± 0.1240) × 10^−5^	0.3507 ± 0.0014
HEA/WC-Nb	−(0.7650 ± 0.0337)	(3.3820 ± 0.0415) × 10^−6^	0.039 ± 0.058

## Data Availability

The original contributions presented in this study are included in the article. Further inquiries can be directed to the corresponding author.
